# Polystyrene-Modulated Polypyrrole to Achieve Controllable Electromagnetic-Wave Absorption with Enhanced Environmental Stability

**DOI:** 10.3390/nano12152698

**Published:** 2022-08-05

**Authors:** Huiling Gu, Ji Huang, Na Li, Hua Yang, Yin Wang, Yang Zhang, Chengjun Dong, Gang Chen, Hongtao Guan

**Affiliations:** 1 School of Materials and Energy, Yunnan University, Kunming 650091, China; 2 Department of Materials Science and Engineering, Beijing Technology and Business University, Beijing 100048, China

**Keywords:** microwave absorption, polypyrrole composite, polystyrene, in situ bulk polymerization

## Abstract

The moderation of the dielectric properties of polymer composites and their environmental stability need to be considered comprehensively in the design of microwave-absorbing materials. In this work, polypyrrole/polystyrene (PPy/PS) composited particles were synthesized through a facile in situ bulk polymerization procedure. The PS component can be modulated conveniently by controlling the polymerization time. FTIR and Raman analyses disclosed that the PS component was immobilized in PPy via covalent bonds. The electromagnetic characterization results indicated that the dielectric properties and, thus, the microwave absorption could be controlled when the styrene polymerization was prolonged from 6 h (PPy-6) to 19 h (PPy-19). The composite PPy-19 displayed an optimal reflection loss of −51.7 dB with a matching thickness of 3.16 mm, and the effective absorption bandwidth (EAB) even reached 5.8 GHz at 2.4 mm. The PS component endowed PPy/PS composites with more robust environmental stability than homogeneous PPy. After being exposed to air for 365 days and hydrothermally treated at 100 °C for 12 h, PPy-19 still exhibited a reflection loss superior to −20 dB. The present work provides a new insight into the adjustment of the electromagnetic properties of PPy composites to fabricate high-performance microwave absorbers with superior environmental stability.

## 1. Introduction

With the rapid development of technology, the widespread use of high-frequency and high-power electrical equipment will inevitably cause electromagnetic interference (EMI) and electromagnetic pollution. This inspires researchers to design and fabricate excellent electromagnetic-wave (EMW)-absorbing materials or microwave-absorbing materials (MAMs) to eliminate the harm brought about by electromagnetic pollution and guarantee the safety of electronic devices [1,2,3]. Generally, two critical indicators are employed to characterize the electromagnetic-absorbing performance of a MAM. Firstly, reflection loss (*RL*) is used to indicate the absorption capacity at a certain frequency range. Furthermore, the effective absorption bandwidth (EAB) is an important parameter, which is the frequency range with a superior *RL* over −10 dB. Currently, both the theoretical and experimental research hotspots of MAMs mainly focus on obtaining microwave-absorbing materials with excellent performance in strong and wide absorption bandwidths. Meanwhile, the weight and thickness should be greatly reduced for advanced applications [4]. For practical applications, however, in addition to the absorption capacity, the service life and absorption capacity under long-term environmental conditions should also be taken into account. Therefore, the pursuit of MAMs with excellent performance and good stability is a practical priority [5].

Among all the MAMs, conductive polymers have attracted extensive interest because of their light specific gravity, good electrical conductivity and facile fabrication conditions [6,7,8,9]. More importantly, by tuning the polymerization process, the conductivity of conducting polymers can be modulated from insulator to conductor [10], which greatly favors the tuning of dielectric loss and impedance to improve its microwave-absorption performance [11,12]. Polypyrrole (PPy) shows an especially promising application as a microwave absorber due to its tunable dielectric constant and electric conductivity through doping, composition, etc. [13,14]. However, the electromagnetic-absorption performance of PPy itself is relatively low due to poor impedance matching or mismatched impedance [15,16]. To date, two typical strategies are generally used to improve the electromagnetic-absorbing performance of PPy. The design of PPy in homogeneous morphology and microstructure is especially highly focused. Ren et al. [17] demonstrated that superhelical chiral PPy nanofibers exhibited excellent microwave-absorption performance, in which the minimum *RL* (*RL*_min_) could reach −44.5 dB with a loading of only 6 wt%. Another effective way is to couple PPy with other dielectric-loss or magnetic materials. Thus, excellent EMW absorption can be achieved due to impedance matching and the synergistic effects on the dissipation of electromagnetic energy [7,18,19]. For instance, PPy coupled with biomass carbon and Ni particles gives an *RL*_min_ of −42 dB with an EAB of 5.24 GHz due to the improved impedance matching and synergistic effects of dielectric and magnetic loss [7].

It should be noted that it is apt for PPy to be oxidized in air, resulting in instability in its physical and chemical properties. Recent studies have confirmed that the presence of an air-oxidized phase results in electrons becoming trapped in adjacent monomer units, structural defects and the disruption of double-bond conjugation in PPy; this, subsequently, leads to a conductivity decrease to deteriorate the microwave-absorption performance [20]. Thus, taking a facile approach is highly required to prevent PPy oxidation in air with good impedance matching. It is reasonable to compound PPy with an electromagnetic transparent polymer to endow PPy with appropriate electrical conductivity, as well as stability, in the environment. Polystyrene (PS) shows good microwave transmittance with a low dielectric constant (2.6–2.8), which makes it an excellent candidate to be used as protective layers with electromagnetic transparent characteristics [21,22]. For example, spherical Ni@C/PS particles exhibited remarkably enhanced microwave absorption with increasing diameters of PS spheres due to improved impedance matching [23]. Moreover, the charge carriers’ accumulation at the inter-sphere interfaces is also greatly attributed to microwave performance arising from interfacial polarization [24,25]. Therefore, the above analysis results provide a clue for the introduction of PS into PPy to achieve excellent microwave absorption and stability for practical application.

In this study, a facile and scalable method for preparing PS/PPy composites is proposed. A dense electromagnetic-transmitting PS barrier was introduced into a PPy absorber through in situ polymerization to obtain PS/PPy composited particles. The microwave transparency of PS is beneficial for the transmission of the incident wave into the interior of the PS/PPy composites and, thus, improves the impedance matching. Instead of connecting through non-reactive groups, PPy is linked with PS by covalent bonds. More dipoles between the PS and PPy are formed. The resulting interfacial polarization and related relaxation exert a positive effect on the overall microwave-absorbing properties. An *RL*_min_ value of −51.7 dB is obtained at 10.2 GHz with a matching thickness of 3.16 mm. By adjusting the reaction time of PS, the dielectric properties of the PS/PPy composites are effectively modulated to regulate their electromagnetic properties. Moreover, the PS component effectively blocks direct contact between air and the inner PPy, thus improving the environmental stability of PPy. The microwave absorption of the PS/PPy composite shows no obvious degradation after being exposed to air for 12 months, which indicates its robust durability. We believe that this strategy will be a promising route for the future optimization of microwave absorbers with high dielectric and low corrosion resistance.

## 2. Materials and Methods

### 2.1. Chemicals and Materials

Pyrrole (Py) and dibenzoyl peroxide (BPO) were purchased from Aladdin Bio-Chem Technology Co., Ltd. (Shanghai, China). Styrene (St), ammonium persulfate (APS) and sodium p-styrene sulfonate (SS) were supplied by Macklin Biochemical Co., Ltd. (Shanghai, China). All of the chemicals were of analytical grade and used without further purification. Freshly prepared deionized water was used throughout the experiment.

### 2.2. Synthesis of PPy/PS Composites

**Figure 1:**
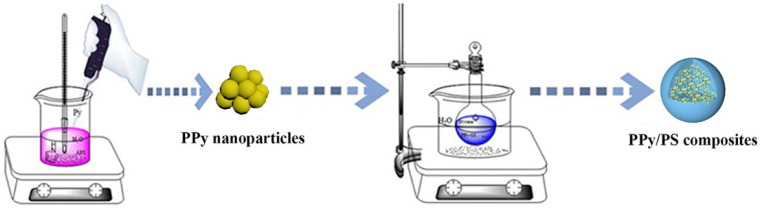
Representative synthetic scheme for the synthesis of PPy/PS.

Typically, a radical bulk-polymerization process is undertaken for the synthesis of a PPy/PS composite. The synthetic route is schematically illustrated in Figure 1. Firstly, 2.0 mL Py monomer and 8.82 g SS were dissolved in 200 mL water for 30 min in an ice-water bath. Then, 40 mL APS solution (1.80 g APS) was added to the above mixture. Polymerization was allowed to proceed for 24 h. The obtained PPy/SS was collected after being rinsed with water and ethanol several times, and then, dried at room temperature.

To avoid dedoping of SS from the PPy/SS, the polymerization process was conducted at separated temperatures of 40 °C and 60 °C for different durations. The mixture containing 0.5 g PPy/SS, 0.10 g BPO and 10.0 g St monomer was reacted at 40 °C for 10 h under stirring at 400 rpm. Then, the mixture was sealed and heated at 60 °C for different durations. Additionally, the as-prepared PPy/PS composites were named as PPy-6, PPy-9, PPy-12, PPy-15 and PPy-19, corresponding to the different reaction times of 6, 9, 12, 15 and 19 h, respectively. For comparison, homogeneous PPy without PS was synthesized and named PPy-0.

### 2.3. Characterization

The functional groups of the PPy/PS composites were examined via Fourier transform infrared spectrometer (FTIR, Thermofisher Bruck Nicolet iS 10, Waltham, MA, USA) using KBr method within the wavenumber range of 400–4000 cm^−1^. Raman spectra were recorded on an inVia spectrometer (Renishaw, London, UK), wherein an excitation wavelength of 514 nm was adopted. Scanning electron microscopy (SEM, FEI Nova Nano SEM 450, Hillsboro, OR, USA) and transmission electron microscopy (TEM, JEOL JEM-2100, Tokyo, Japan) were used to characterize the morphology and microstructures. A thermogravimetric (TG) analysis was carried out using a thermogravimetric analyzer (TGA, TA Instruments SDT-2960, New Castle, DE, USA) from room temperature to 800 °C with a N_2_ flow at a heating rate of 10 °C min^−1^.

The electromagnetic-parameter measurements were carried out via a vector network analyzer (VNA, Keysight P5004A, Santa Rosa, CA, USA) in the frequency range of 2–18 GHz. Firstly, a mixture was formed by uniformly mixing the as-prepared PPy/PS composites with 30 wt.% paraffin. Then, it was pressed into a torus of 7.00 mm and 3.04 mm in outer and inner diameter, respectively, as well as a thickness of 2 mm.

Finally, the microwave-absorbing properties of the samples were evaluated through the electromagnetic parameters based on the transmission line (TML) theory.

## 3. Results and Discussion

### 3.1. Structural, Morphology and Component Analysis

**Figure 2:**
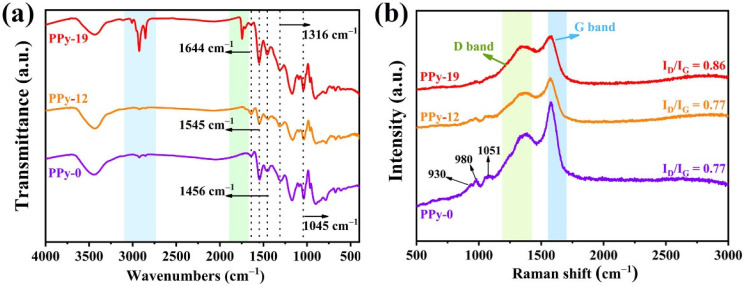
FTIR (**a**) and Raman (**b**) spectra of PPy-0, PPy-12 and PPy-19.

As shown in Figure 2a, two characteristic peaks located at 1644 cm^−1^, 1545 cm^−1^ and 1456 cm^−1^ are attributed to the stretching vibration of the C–C bond in the aromatic ring and pyrrole ring. In addition, the peak at 1456 cm^−1^ is associated with the stretching vibration of C–N bond. The peaks at 1316 cm^−1^ and 1045 cm^−1^ correspond to the in-plane vibration absorption peaks of the conjugated C−N and C–H bonds [13]. Significantly, compared with PPy-0 and PPy-12, the characteristic peaks at 2920 cm^−1^ and 2848 cm^−1^ that appear for PPy-19 are attributed to the asymmetric and symmetric stretching vibrations of –CH_2_–, which are caused by the copolymerization between PS and PPy [26,27]. Meanwhile, the typical adsorption peaks at 1700−1600 cm^−1^ originate from the stretching vibration of the C = C bond of the benzene ring, indicating the existence of a PS component [28].

To further reveal the chemical structure of the PPy/PS composites, a Raman measurement was performed. The corresponding Raman spectra are shown in Figure 2b. Clearly, all the characteristic peaks are reserved after the compound with PS, indicating the preservation of the PPy chemical structure in the composites. The peaks appear at around 1580 cm^−1^ and 1366 cm^−1^ for pure PPy, and the PPy/PS composites are called D-band and G-band, respectively; these are from the π conjugated structure and the ring-stretching mode of the polymer backbone [26]. The peak located at about 1051 cm^−1^ can be ascribed to the C–H in-plane deformation. In addition, the other two peaks at about 930 cm^−1^ and 980 cm^−1^ are associated with the quinoid polaronic and bipolaronic structure [29]. With the prolonging of the reaction time, the contents of PS in the composites increase gradually, leading to a reduction in the characteristic peaks of PPy. It is widely accepted that the D-band and G-band originate from the disordered and in-plane vibrations in sp^3^ and sp^2^ carbon atoms, respectively [28]. A higher value of *I*_D_/*I*_G_ indicates more crystal defects of the C atom in PPy [29]. The *I*_D_/*I*_G_ ratios of PPy-0, PPy-12 and PPy-19 are estimated to be 0.77, 0.77 and 0.86, respectively. A higher *I*_D_/*I*_G_ ratio of PPy-19 indicates richness in its structural defects and disorders due to the introduction of PS in the reduction process. The defects and disorders will result in higher attenuation of the incident electromagnetic energy, which is favorable for EMW absorption of the absorbers [3].

To further determine the relative composition of the PPy/PS composite, TGA analysis was performed for the samples PPy-0, PPy-12 and PPy-19 under a N_2_ atmosphere, as shown in Figure S1. Herein, the first slight weight loss (about 2–9 wt%) under 150 °C is observed because of the evaporation of adsorbed water and the solvent molecules. It is known that PPy is hygroscopic and water evaporates during the heating process, which causes a mass-loss of about 8.4 wt.% in a temperature region below 150 °C [30]. The evaluated mass-loss of PPy-12 and PPy-19 during heating to 150 °C is only 4.2 wt.% and 2.8 wt.%, suggesting a drop in PPy’s affinity towards humidity after polymerization with PS. In a temperature range from 150 °C to 300 °C, the PPy/PS composite exhibits distinct weight loss (about 15%) compared with PPy-0 due to the decomposition of the PS oligomers and the grafted copolymer of St and SS [31,32]. As the temperature increases under an inert atmosphere, the main mass-loss of PPy starts at about 300 °C, corresponding to PPy degradation [33]. In terms of weight changes, the mass-percentage of PS in samples PPy-12 and PPy-19 is estimated to be about 10% and 31%, respectively. It is evident that the contents of PS nanoparticles in the as-synthesized PPy/PS samples increase gradually during the polymerization process.

Based on the structural changes revealed by FTIR, Raman and TG, a possible formation mechanism for the PPy/PS composite is proposed. To enhance the compatibility between PPy and PS, SS with C=C was used for the doping of PPy, where part of the PS was covalently connected to PPy. It is known that SS molecules may be dedoped from PPy-SS, so to avoid the dedoping of PPy, radical bulk polymerization was conducted under a temperature gradient [31]. An appreciable content of PS was grafted onto PPy crystals by heating them in styrene at 60 °C for 12 h. The PPy was copolymerized with styrene under the action of an initiator, and PPy/PS was, thus, obtained through conjugated covalent bonds.

### 3.2. Morphological Analysis

**Figure 3:**
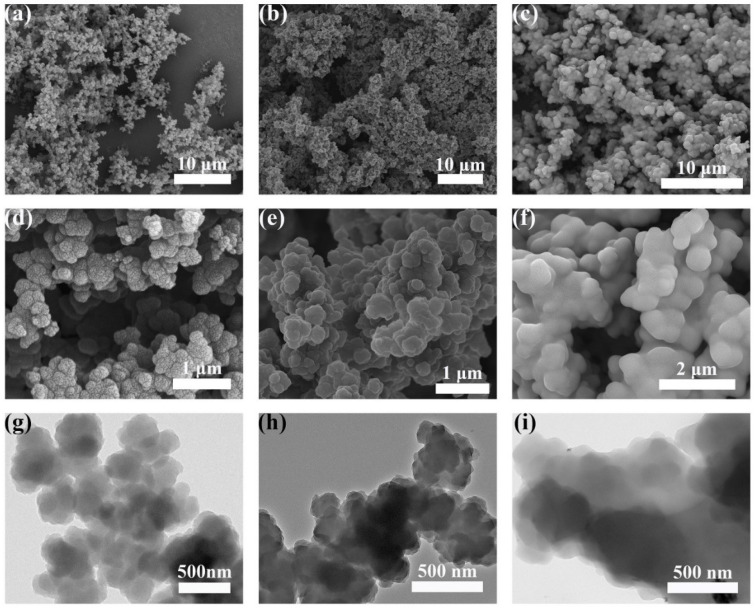
SEM (**a**–**f**) and TEM (**g**–**i**) micrographs of the PPy-0 (**a**,**d**,**g**), PPy-12 (**b**,**e**,**h**) and PPy-19 (**c**,**f**,**i**).

The evolution of morphologies with the PS compound was disclosed via SEM and TEM. The micrograph of PPy (Figure 3a) exhibits a rough surface, and some tiny particles are found to be collected together to form a sphere-like structure, as shown in Figure 3d. 

However, the surface morphology of the PPy/PS composite particles gradually changes from rough to smooth with the addition of PS (Figure 3b,c). In Figure 3e, an irregular smooth morphology is found as a result of the PS covering. As the reaction between the PS and PPy continues, the composite PPy-19 forms a capsule structure, whereby PS is coated on the surface of PPy; this is shown clearly in Figure 3f. Further, the TEM image in Figure 3i indicates that PPy-19 displays complete encapsulation of PPy nanoparticles by PS, compared with the pure PPy-0 in Figure 3g.

### 3.3. Microwave-Absorption Property

According to the electromagnetic theory, the microwave-absorption property of a MAM is particularly sensitive to its complex relative permittivity (*ε*_r_ = *ε*′−*jε*″) and complex relative permeability (*μ*_r_ = *μ*′−*jμ*″) [3]. Here, the real parts (*ε*′ and *μ′*) present the storage ability, while the imaginary parts (*ε″* and *μ*″) stand for the loss property (or dissipation) of the incident electromagnetic energy, respectively [34]. Correspondingly, the dielectric-loss tangents (tan*δ*_e_ = *ε*″/*ε*′) and magnetic loss tangents (tan*δ*_m_ = *μ*″/*μ*′) are defined in order to evaluate power loss in the MAM with respect to the stored power. Since PPy and PPy/PS composites are nonmagnetic materials, their magnetic permeability shows similar values with a *μ*′ close to 1 and a *μ*″ close to 0. Therefore, in the present work, the complex dielectric properties are analyzed. Correspondingly, the dielectric-loss factors are suggested in order to evaluate the attenuation performance and loss mechanism.

**Figure 4:**
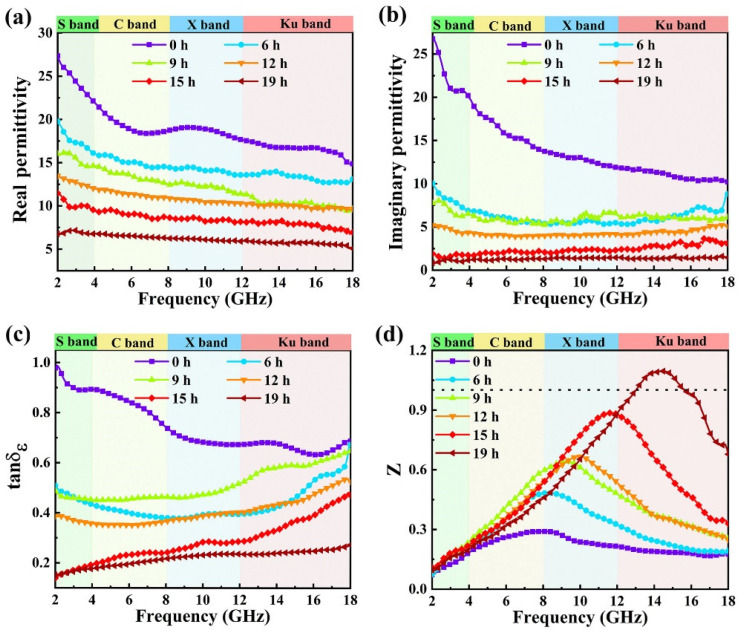
Frequency dependence of the real (**a**) and imaginary (**b**) parts of permittivity, dielectric loss tan*δ*_e_ (**c**) and normalized characteristic impedance *Z* at a thickness of 2.0 mm (**d**) for the composites.

Figure 4a,b present variations in the complex permittivity with varying frequency for PPy-0, PPy-6, PPy-9, PPy-12, PPy-15 and PPy-19, respectively. It should be pointed out that the introduction of PS into PPy leads to a decrease in *ε*′ and *ε*″ [10]. It can be clearly seen that PPy-0 exhibits the highest *ε*′ and *ε*″, with their values changing from 27.4 to 14.9 and 17.6 to 11.1, respectively, with the frequency rising. With the introduction of PS into PPy, *ε*′ and *ε*″ decrease significantly compared to those of homogeneous PPy. Moreover, both *ε*′ and *ε*″ illustrate a declining trend with increasing polymerization time. In detail, the frequency-dependent *ε*′ values of PPy-6, PPy-9, PPy-12, PPy-15 and PPy-19 decrease from 19.9 to 12.8, 16.2 to 9.7, 13.3 to 9.6, 11.6 to 6.8 and 6.7 to 5.1, respectively, when the frequency rises from 2 GHz to 18 GHz. Similarly, the *ε*″ values of the composites gradually vary from 10.1 to 8.7, 7.8 to 6.0, 5.1 to 5.2, 2.0 to 3.2, and 0.8 to 1.6, respectively, with increasing frequency.

Furthermore, the dielectric-loss properties of the PPy/PS composites and PPy were evaluated through their dielectric-loss tangents. From Figure 4c, it is shown that the tan*δ*_e_ values of most composites exhibit decreasing trends with increasing reaction time, except that PPy-9 provides a larger tan*δ*_e_ than PPy-6, due to its larger *ε*″ and smaller *ε*′ compared to that of PPy-6. The tan*δ*_e_ values of PPy-15 and PPy-19 are lower than 0.5, especially in the low frequency range. Specifically, PPy-19 has a much smaller tan*δ*_e_ than homogeneous PPy, indicating that the dielectric loss of homogeneous PPy makes a greater contribution to microwave absorption than that of PPy-19.

In addition to the loss tangent, impedance matching (*Z*) is another crucial parameter in determining microwave-absorption properties. *Z* can be expressed as *Z* = |*Z*_in_/*Z*_0_|, in which *Z*_0_ (120π Ω) represents the impedance of free space. *Z*_in_ is the input impedance of the MAM and can be expressed as follows [3]:

(1) $$Z_{\mathrm{in}}=Z_{0}\sqrt{\frac{\mu_{r}}{\varepsilon_{r}}}\tanh\left(j\frac{2\pi f}{c}d\sqrt{\mu_{r}\varepsilon_{r}}\right)$$

where *f* is the frequency, *c* is the speed of the electromagnetic wave in free space, and *d* is the thickness of the sample.

From Equation (1), it is clear that when *Z*_in_ is close to *Z*_0_, the value of *Z* is close to 1. The incident electromagnetic wave will easily propagate into the absorber [35]. When *Z*_in_ equals *Z*_0_, i.e., *Z* = 1, it is called ideal impedance matching. Thus, a higher dielectric loss (larger than 0.5) will be detrimental to the impedance-matching characteristic of the MAM. Figure 4d shows the variation of *Z* with varying frequency for the as-prepared samples at a thickness of 2.0 mm. It is observed that for the PPy-19 composite, *Z* = 1 was obtained at 12.9 GHz and 15.6 GHz, which implies its better impedance-matching characteristics at these two frequencies. Accordingly, impedance-matching should be considered in combination with loss performance in a MAM design. Combined with its dielectric loss, it can be expected that PPy-19 may possess enhanced microwave-absorption properties.

Based on the electromagnetic parameters in Figure 4, the microwave-absorption properties of the composites were calculated based on the TML theory using the following Equation (2):

(2) $$RL=20\log\left|\frac{Z_{\mathrm{in}}-Z_{0}}{Z_{\mathrm{in}}+Z_{0}}\right|$$

**Figure 5:**
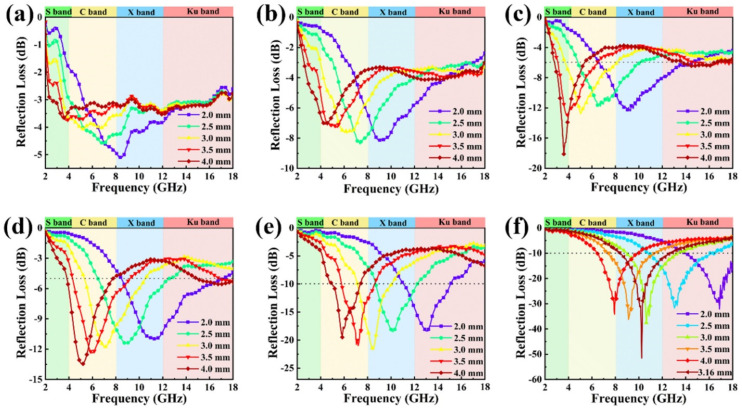
Reflection loss versus frequency and thickness for PPy-0 (**a**), PPy-6 (**b**), PPy-9 (**c**), PPy-12 (**d**), PPy-15 (**e**) and PPy-19 (**f**).

Figure 5 displays the *RL* variations with varying frequency of the PPy/PS composites at different thicknesses with a filling ratio of 30 wt.% in the paraffin matrix. Generally, *RL* with a value superior to −10 dB is suitable for practical applications, in that 90% of the incident microwave energy can be attenuated in this situation. In addition, the frequency range in which the *RL* is superior to −10 dB is called the effective absorption bandwidth (EAB). With an increase in thickness, the peaks of reflection loss move to a lower frequency, as shown in Figure 5; this is consistent with the formula *f* = *c*/(2*πμ*″*d*) (*f*, *μ*″ and *d* are the resonant frequency, imaginary permeability and the thickness of the samples, respectively.) [36]. With increasing polymerization time, the PS contents in the PPy/PS composites increase and the *RL*_min_ values become greater, which proves the enhancement of microwave absorption for the PPy/PS composites. As is in Figure 5a, pure PPy only gives an *RL*_min_ of −5.1 dB at 8.2 GHz due to its high permittivity. Regarding sample PPy-6 in Figure 5b, it exhibits an *RL*_min_ of −8.3 dB at 7.5 GHz, with a thickness of 2.5 mm, while the *RL*_min_ for PPy-19 in Figure 5f reaches −51.7 dB at 10.2 GHz, with a matching thickness of 3.16 mm. When its matching thickness is tuned to 2.4 mm, its *RL*_min_ is also over −40 dB. Moreover, its EAB reaches as high as 5.8 GHz (11.28−17.09 GHz). From Figure 5d, it is apparent that the impedance-matching characteristic of sample PPy-19 is much better than that of PPy-0, which results in its excellent microwave property. It is easy for the incident electromagnetic wave to enter into PPy-19, rather than being reflected at the surface. The absorption ability of PPy-19, thus, could be ascribed to both its good impedance-matching characteristics and its adequate attenuation ability, which are caused by dielectric polarization and interfacial polarization. 

**Figure 6:**
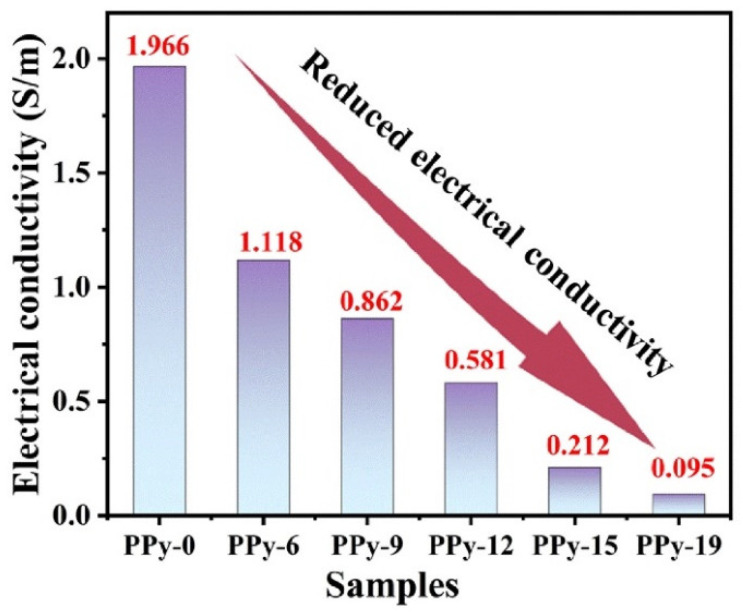
The electrical conductivity of the PPy/PS composites.

The dielectric loss of a microwave absorber is mainly determined by polarization loss and conductance loss, while polarization loss is generally determined by dipole polarization, interfacial polarization and their related relaxation. The conductance loss of as-synthesized PPy/PS composites mainly stems from the contribution of PPy, due to its much higher conductivity than pure PS. When introducing PS to PPy, the conductivity of the originally flowing electrons is greatly reduced. Due to the electrical insulating properties of PS, the electrical conductivity of the PPy/PS composites is greatly reduced. As a result, the high conductivity of PPy is moderated and appropriate permittivity is achieved [19,37,38]. Figure 6 shows the electrical conductivity (*σ*) of PPy and the PPy/PS composites. It can be clearly seen that PPy has a high conductivity value of 1.966 S/m. After PS is incorporated, the conductivity values of the composites reduce greatly. In addition to the conduction loss, the potential difference between PPy and PS causes charge accumulation at the interface under an electromagnetic field and intensifies the polarization relaxation, which is helpful for dielectric loss [39]. Additionally, the existence of a large number of inner and outer surfaces and interfaces in PPy/PS composites will create interfacial polarization to improve the dielectric loss. 

To explore the contribution of polarization, the Debye relaxation theory is used, which is plotted as a Cole–Cole semicircle in the following [3]:

(3) $${\left(\varepsilon^{\prime }-\frac{\varepsilon_{s}+\varepsilon_{\infty }}{2}\right)}^{2}+{\left(\varepsilon^{\prime \prime }\right)}^{2}={\left(\frac{\varepsilon_{s}-\varepsilon_{\infty }}{2}\right)}^{2}$$

**Figure 7:**
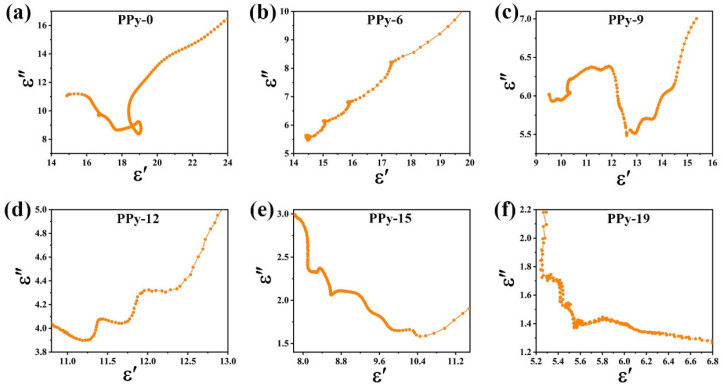
The Cole–Cole curves for PPy-0 (**a**), PPy-6 (**b**), PPy-9 (**c**), PPy-12 (**d**), PPy-15 (**e**), and PPy-19 (**f**).

Here, *ε_s_* and *ε*_∞_ stand for the static dielectric constant and the dielectric constant at infinite frequency. *τ* refers to the polarization relaxation time. Figure 7 shows the Cole–Cole plots of the composites. Several semicircles for each composite are observed, indicating the complicated polarization mechanisms in the PPy composites. The dipole pairs could accumulate at the interfaces to cause dipole polarization and interface polarization. At the end of each plot, there is a straight line, indicating the contribution of conduction loss to the electromagnetic attenuation [40].

### 3.4. Environmental Stability of Microwave Absorption

**Figure 8:**
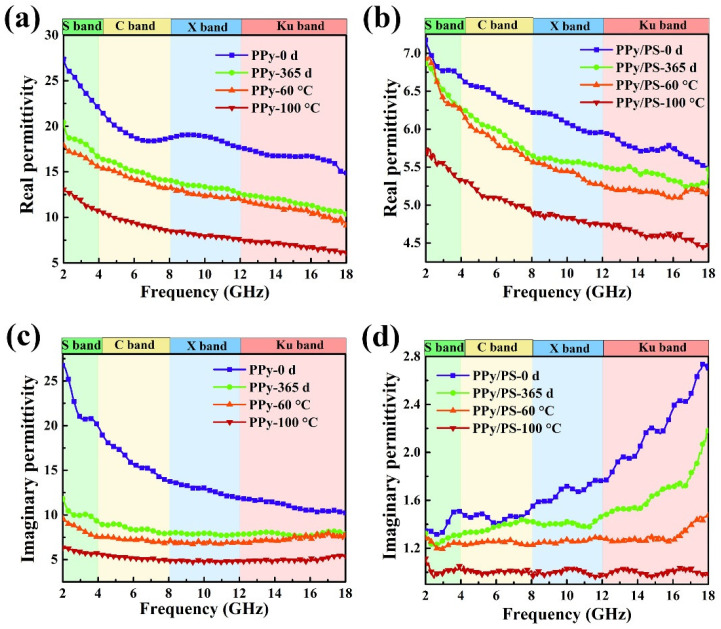
The real (**a**,**b**) and imaginary (**c**,**d**) permittivity of PPy (**a**,**c**) and PPy-19 (**b**,**d**) under different environmental conditions.

As is well known, doped PPy is not environmentally stable and is easily dedoped. To verify the effects of incorporated PS on the electromagnetic stability of the PPy/PS composites, PPy-19 was taken as a candidate and its environmental stability for electromagnetic parameters was examined after placing it at room temperature for 365 days. Figure 8 demonstrates the electromagnetic parameters of pure PPy and the PPy-19 composite after being treated under different conditions. From Figure 8a,c, both the real and imaginary parts of permittivity reduce significantly after 365 days in a natural environment. For example, the *ε*′ of pure PPy at 2 GHz decreased from 27.35 to 20.42 with a drop of 25.34% after 365 days in the air, while its *ε*′ at 18 GHz dropped from 14.87 to 10.35 with a 30.40% decline. On the contrary, after being coated with PS, the *ε*′ of PPy/PS only shows a slight reduction from 7.17 to 6.87 at 2 GHz, and from 5.49 to 5.47 at 18 GHz, as is shown in Figure 8b. The reduction in *ε*″ after 365 days in the air is also alleviated for PPy/PS composites compared with PPy-0 (Figure 8d).

Doped PPy can exhibit high electrical conductivity, but at the expense of its chemical stability. It is easy for doped PPy to be oxidized in air, which leads to a decrease in its conductivity and, thus, its dielectric permittivity. Nevertheless, after being incorporated with PS, the PS component can protect the interior PPy from further oxidation and, thus, alleviate the decreasing trend of dielectric permittivity. To further clarify the environmental stability of PPy/PS composites, the dielectric properties of PPy-0 and PPy-19 are also examined at high temperature through sealing in a PTFE-lined autoclave at 60 °C for 24 h and 100 °C for 12 h, respectively. From Figure 8, it is obvious that both the *ε*′ and *ε*″ of PPy-0 and PPy-19 decrease with hydrothermal treatment. The downward trend is similar to that of the 365-day exposure in the air. However, after the samples are hydrothermally treated at 100 °C for 12 h, both their *ε*′ and *ε*″ values deteriorate pronouncedly. This phenomenon can be ascribed to the dedoping of PPy at high temperatures. SEM characterization was carried out to check the encapsulation of PS after the sample PPy-19 was treated at 100 °C for 12 h. From Figure S2 it can be seen that the PS shell is broken and the PPy has been exposed to air. Since the PS shell obtained at a low temperature (60 °C) has relatively low polymerization and could not stand such a high temperature as 100 °C, it would be destroyed under hydrothermal treatment.

**Figure 9:**
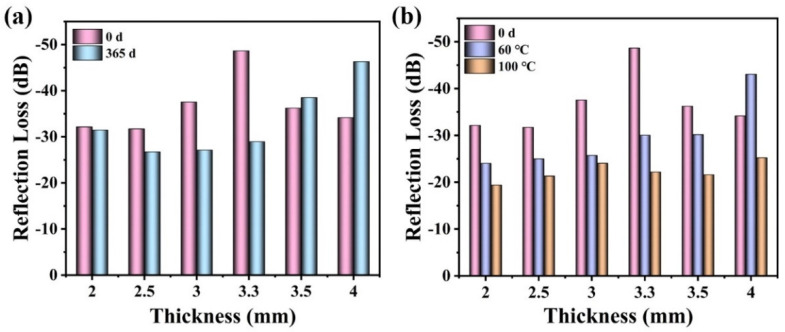
The absorption performance of PPy-19 under different environmental conditions. (**a**) Exposed to air for 365 days; (**b**) Hydrothermally treated at 60 °C for 24 h and 100 °C for 12 h, respectively.

Figure 9 demonstrates the stability of the microwave absorption of PPy-19 after different environment treatments. It can be clearly seen that the absorption performance exhibits a decreasing trend after exposure to air or to a high temperature, especially after being treated hydrothermally at 100 °C for 12 h, as in Figure 9b, whereby its microwave absorption is reduced by approximately 50%. However, the *RL* is still superior to −20 dB at most thicknesses, indicating that PPy-19 has excellent microwave-absorption performance, even under a harsh environment.

**Figure 10:**
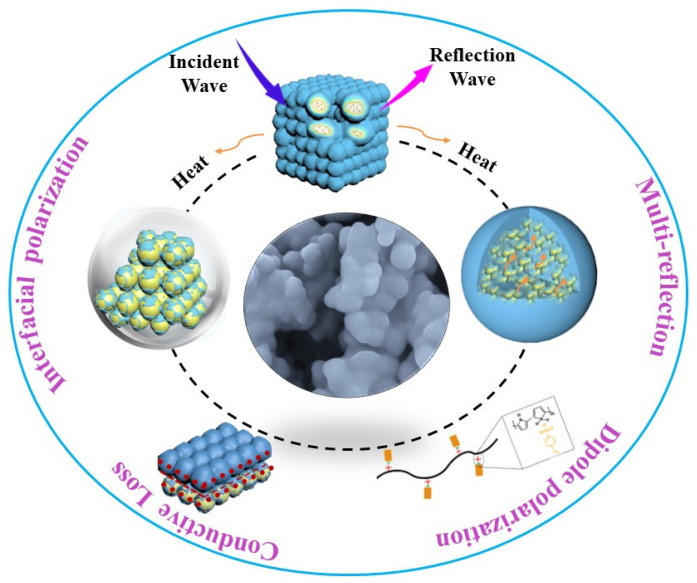
An illustrative diagram for the microwave-absorption mechanisms in PPy/PS composites.

In terms of the above-mentioned analyses, a possible microwave-absorption mechanism is schematically elucidated in Figure 10. The enhanced microwave-absorption properties of the PPy/PS composite can be ascribed to the following factors: Firstly, the abundant interfaces and junctions between PS and PPy components induce rich bound charges to give birth to interfacial polarization and the related relaxation. The sufficient surfaces and interfaces also bring about multiple-reflection of the incident microwave, which favors microwave attenuation [4]. Secondly, the covalent connection between PPy and PS plays roles in increasing transfer channels as well as promoting electron polarization. Moreover, the covalent connection increases the nonuniform distribution of electron density in PPy to enhance the synergistic effects of PPy and PS [41,42]. Thirdly, the defects and functional groups can lead to surface-charge redistribution and result in the corresponding dipole polarization and related relaxation. Finally, the addition of PS modifies the dielectric loss of the PPy/PS composites, so that the impedance matching is improved based on the high attenuation coefficient [43].

## 4. Conclusions

This work presents a generalizable strategy for the immobilization of PPy within PS via facile in situ bulk polymerization. The as-synthesized PPy/PS nanocomposites exhibit superior microwave-absorption properties. Here, PPy-19 displays an optimal reflection loss of −51.7 dB at a matching thickness of 3.16 mm and possesses a broader effective absorption bandwidth reaching 5.8 GHz at 2.4 mm. It is worth noting that the microwave-absorption performance can be easily tuned by changing the PS concentration in the composites. The enhanced microwave-absorption property of PPy/PS composites is attributed to the improved impedance-matching and synergistic effect. Moreover, the microwave-absorption properties of the obtained composites exhibit superior environmental stability. In summary, this study provides an efficient method for modulating the electromagnetic properties of PPy composites and, thus, presents a new strategy for fabricating microwave absorbers with high absorption performance.

## Data Availability

The data presented in this study are available on request from the corresponding author.

## Supplementary Materials

The following supporting information can be downloaded at: https://www.mdpi.com/article/10.3390/nano12152698/s1, Figure S1: TG curves of PPy-0, PPy-12 and PPy-19 samples; Figure S2: SEM of PPy-19 after hydrothermal treatment at 100 °C for 12 h.
