# Supplementary material for: Polystyrene-Modulated Polypyrrole to Achieve Controllable Electromagnetic-Wave Absorption with Enhanced Environmental Stability
Source: Nanomaterials (Basel). 2022 Aug 5;12(15):2698. doi: 10.3390/nano12152698 (PMC9370624; doi:10.3390/nano12152698)
Supplement: Supplementary file 1 [file nanomaterials-12-02698-s001.zip › nanomaterials-1810174-supplementary.pdf]

nanomaterials

MDPI

Supporting Information

Polystyrene-Modulated Polypyrrole to Achieve Controllable Electromagnetic-Wave Absorption with Enhanced Environmental Stability

Huiling Gu [1], Ji Huang [1], Na Li [1], Hua Yang [1], Yin Wang [1], Yang Zhang [2,*], Chengjun Dong [1], Gang Chen [1] and Hongtao Guan [1,*]

1 School of Materials and Energy, Yunnan University, Kunming 650091, China; guhl@mail.ynu.edu.cn (H.G.); 12020135002@mail.ynu.edu.cn (J.H.); 12020235076@mail.ynu.edu.cn (N.L.); yh1223g1@mail.ynu.edu.cn (H.Y.); wangyin@mail.ynu.edu.cn (Y.W.); dongcj@ynu.edu.cn (C.D.); chengangkm@ynu.edu.cn (G.C.)

2 Department of Materials Science and Engineering, Beijing Technology and Business University, Beijing 100048, China

* Correspondence: zhyang@iccas.ac.cn (Y.Z.); htguan06@ynu.edu.cn (H.G.)

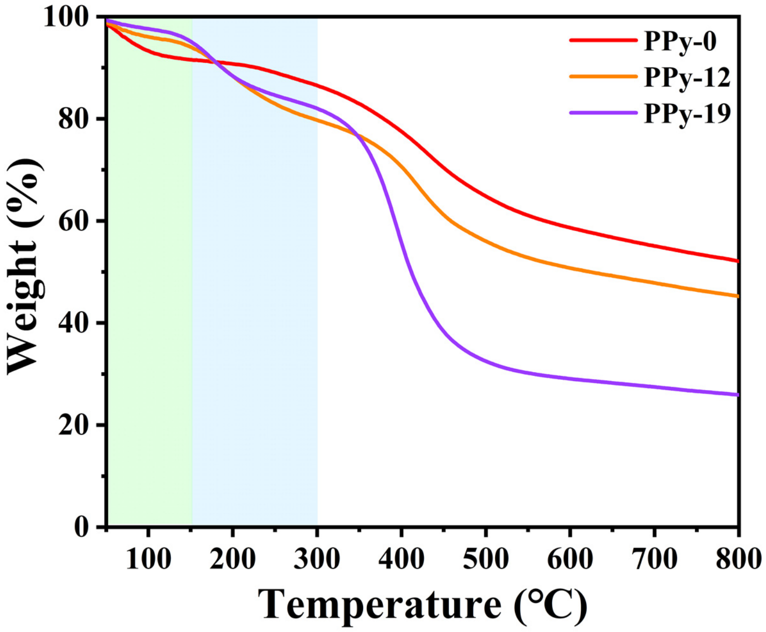

Figure S1. TG curves of PPy-0, PPy-12 and PPy-19 samples.

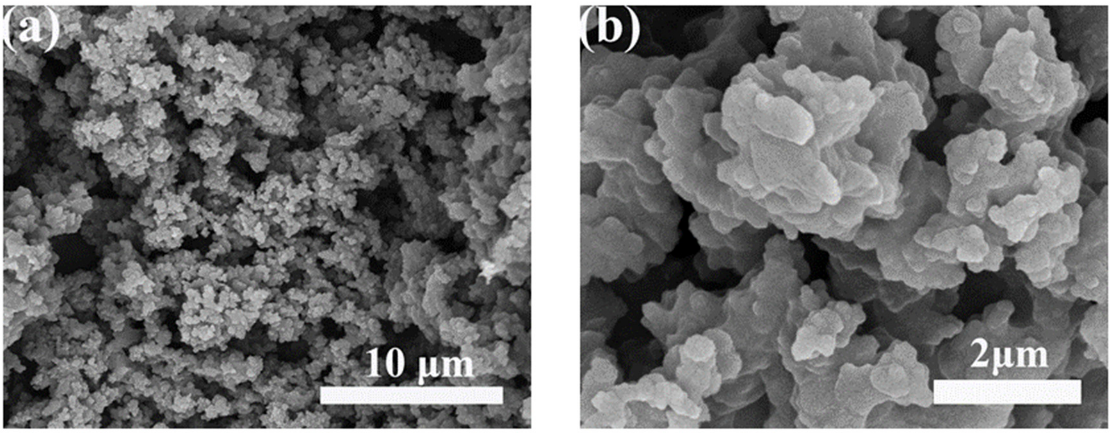

Figure S2. SEM of the PPy-19 after a hydrothermal treatment at 100 ºC for 12 h.
